# Quality of patient- and proxy-reported outcomes for children with impairment of the upper extremity: a systematic review using the COSMIN methodology

**DOI:** 10.1186/s41687-022-00469-4

**Published:** 2022-06-02

**Authors:** J. P. Ruben Kalle, Tim F. F. Saris, Inger N. Sierevelt, Denise Eygendaal, Christiaan J. A. van Bergen

**Affiliations:** 1 Utrecht University, Heidelberglaan 8, 3584CS Utrecht, The Netherlands; 2 Amphia Hospital, Molengracht 21, 4818CK Breda, The Netherlands; 3 Stichting SCORE, Laarderhoogtweg 12, 1101AE Amsterdam, The Netherlands; 4 Erasmus MC, Dr. Molewaterplein 40, 3015GD Rotterdam, The Netherlands; 5 Amphia Hospital, Molengracht 21, 4818CK Breda, The Netherlands

**Keywords:** Pediatric orthopedics, Upper extremity, Measurement properties, Review (publication type), COSMIN

## Abstract

**Background:**

As patient-reported outcome measures (PROMs) have become of significant importance in patient evaluation, adequately selecting the appropriate instrument is an integral part of pediatric orthopedic research and clinical practice. This systematic review provides a comprehensive overview of PROMs targeted at children with impairment of the upper limb, and critically appraises and summarizes the quality of their measurement properties by applying the COnsensus-based Standards for selection of health Measurement INstruments (COSMIN) methodology.

**Methods:**

A systematic search of the MEDLINE and EMBASE databases was performed to identify relevant publications reporting on the development and/or validation of PROMs used for evaluating children with impairment of the upper extremity. Data extraction and quality assessment (including a risk of bias evaluation) of the included studies was undertaken by two reviewers independently and in accordance with COSMIN guidelines.

**Results:**

Out of 6423 screened publications, 32 original articles were eligible for inclusion in this review, reporting evidence on the measurement properties of 22 self- and/or proxy-reported questionnaires (including seven cultural adaptations) for various pediatric orthopedic conditions, including cerebral palsy (CP) and obstetric brachial plexus palsy (OBPP). The measurement property most frequently evaluated was construct validity. No studies evaluating content validity and only four PROM development studies were included. The methodological quality of these development studies was either ‘doubtful’ or ‘inadequate’. The quantity and quality of the evidence on the other measurement properties of the included questionnaires varied substantially with insufficient sample sizes and/or poor methodological quality resulting in significant downgrading of evidence quality.

**Conclusion:**

This review provides a comprehensive overview of currently available PROMs for evaluation of the pediatric upper limb. Based on our findings, none of the PROMs demonstrated sufficient evidence on their measurement properties to justify recommending the use of these instruments. These findings provide room for validation studies on existing pediatric orthopedic upper limb PROMs (especially on content validity), and/or the development of new instruments.

**Supplementary Information:**

The online version contains supplementary material available at 10.1186/s41687-022-00469-4.

## Introduction

Over the last decades, the focus of clinical research has shifted from conventional survival and disease outcomes, to patient experience and patient-reported outcomes (PROs) [1]. A PRO is any report coming directly from a patient, without interpretation by a physician or others, describing the patients’ current health condition [2]. PROs as a primary or secondary outcome can provide a more holistic and comprehensive assessment when investigating the harms and benefits of an intervention [1, 3]. PROs are measured using patient-reported outcome-measures (PROMs), which are the instruments or tools utilized to evaluate the patients’ health status from the patient’s perspective [1, 2].

Orthopedic injuries of the upper extremities are amongst the most common injuries in the pediatric population [4, 5]. As these ailments can be associated with consequential complications and functional disabilities, adequately evaluating patients during follow-up is essential [6]. In recent years, the previously described transition in outcome-focus has also made its way into the rapidly expanding research field of pediatric orthopedics. This shift is reflected by a significant increase in the utilization of PROMs in pediatric orthopedic studies [7–9]. However, an increase in PROM use does not necessarily translate to improved outcome assessment. The misuse of PROMs may prompt researchers to interpret results incorrectly and potentially make misleading or even harmful recommendations for clinical practice [10]. Thus, selecting the appropriate instrument for the appropriate study population and purpose is essential for the further development of PRO-based research [11].

Systematic reviews of PROMs play an important role in guiding PROM selection [12]. By providing an evidence-based overview of available PROMs and presenting recommendations for their use, reviews of PROMs enable clinicians and researchers to find the most suitable instrument for a given purpose [13]. However, to our knowledge, previously published reviews of pediatric orthopedic PROMs either exclusively cater a niche subgroup of patients, or focus on frequency of use, and do not aid in PROM selection [7–9, 14].

As a result, the inadequate application and selection of PROMs is still common practice in pediatric orthopedics. In a recent publication, Arguelles et al. [9] demonstrated that researchers are faced with major challenges when selecting appropriate PROMs. Approximately three quarters of pediatric orthopedic studies reporting PROMs used at least one PROM that was inadequately validated for the population of interest [9]. The improper use of PROMs in pediatric orthopedic research uncovers an urgent need for guidance on PROM selection and application, so that future results can be interpretated adequately and PROMs can be implemented in daily practice with true scientific justification.

Thus, we conducted a systematic review of pediatric orthopedic PROMs validated for children with impairment of the upper extremity. The primary goal of this review was to provide a comprehensive overview of self- and/or proxy-completed questionnaires targeted at children with impairment of the upper limb, and to critically appraise and summarize the quality of their measurement properties. The secondary goal of this review was to provide evidence-based recommendations for PROM selection in pediatric orthopedic research and clinical practice.

## Methods and materials

### Design

In conducting this systematic review, the updated COnsensus-based Standards for selection of health Measurement INstruments (COSMIN) methodology for systematic reviews of PROMs was used [15–17]. This systematic review adhered to the newly revised Preferred Reporting Items for Systematic Reviews and Meta-analyses (PRISMA) statement [18].

### Pre-registration

This study was pre-registered in PROSPERO (PROSPERO registration number: CRD42021254791).

### Search strategy

To identify relevant studies, MEDLINE was systematically searched using PubMed, and EMBASE was systematically searched through the Embase search engine. The timeframe was defined as 1st of January 2000 to 8th of February 2021. The search was restricted to English and/or Dutch articles only by using language filters.

A comprehensive search strategy was constructed in collaboration with a clinical librarian to guarantee a thorough approach. The search strings for each database can be found in full detail in Additional file 1: Appendix 1. The search was initially constructed for PubMed and subsequently adapted to fit the Embase search engine. The search consisted of four distinct elements: (A) search terms describing the population of interest with a validated pediatric study search filter by Leclerq et al. [19], (B) the comprehensive PROM-filter developed by the PROM Group of the University of Oxford, and two validated filters by Terwee et al. [20]: (C) a highly-sensitive measurement property filter and (D) an exclusion filter.

### Eligibility criteria

Articles were considered eligible for inclusion if a full-text original version of the article was available and if the article reported on studies describing the development and/or the evaluation of one or more measurement properties of a generic and/or disease-specific patient-reported and/or proxy-reported questionnaire of any language, in a population consisting of children (0–18 years old) with an orthopedic diagnosis in the upper extremity region. Exclusion criteria consisted of any study design in which the patient-reported and/or parent-proxy-reported questionnaire was only used as an outcome measurement instrument (e.g., randomized controlled trials, longitudinal studies) and/or in which one or more questionnaires were evaluated that aimed to assess the use of prostheses by children (0–18 years old).

### Study selection

First, all eligible studies were selected by screening the title and abstract. Thereafter, all selected papers were screened based on full text. During both phases two reviewers (JPR and TFF) independently identified eligible studies according to the predefined eligibility criteria and afterwards discussed the results. Disagreements were resolved by a third reviewer (IN or CJA). The references of the articles selected for full-text review were thoroughly screened to identify additional citations.

### Data extraction and appraisal

The studies on measurement properties included in this review were assessed in accordance with the extensive and recently improved COSMIN methodology for qualitatively evaluating studies on PROMs [15]. Detailed information on the COSMIN taxonomy, the stepwise approach of the COSMIN methodology and the COSMIN checklists applied in this review, can be found in the corresponding publications by Mokkink et al. [16, 21], Prinsen et al. [15], and Terwee et al. [17].

#### Evaluation of study methodological quality

The COSMIN Risk of Bias checklist [16] was used to rate studies evaluating validity (structural validity, hypotheses testing for construct validity and cross-cultural validity), reliability (internal consistency, reliability and measurement error) and/or responsiveness of a PROM. This modular tool consists of ‘boxes’ containing standards for rating the quality of a study on a measurement property on a four-point rating scale: ‘very good’, ‘adequate’, ‘doubtful’ or ‘inadequate’ [16]. “The worst score counts” principle was then applied to come to an overall methodological quality rating for each individual study on a measurement property [15].

Studies on content validity (content validity and PROM development) were evaluated using the separate COSMIN methodology for evaluating content validity [17]. The quality of these studies was rated following the standards included in the ‘boxes’ of the COSMIN content validity checklist [17]. The worst score counts principle was then used to come to an overall quality rating for the studies [17].

#### Data extraction

Following the methodological quality assessment, data on the characteristics of the included study populations (e.g., sample size, age range, diagnoses), characteristics of the studied PROMs and results of each study on a measurement property were extracted using tables provided by the COSMIN initiative [15].

#### Assessment of psychometric properties

The result of each study on a measurement property was rated against the updated criteria for good measurement properties [15]. The individual results were rated as ‘sufficient’ ( +) when the results were in line with the COSMIN criteria, and ‘insufficient’ (–) if the results did not meet the criteria. The result of a study on a measurement property was considered ‘indeterminate’ (?) when essential information was missing, no hypotheses were defined prior to starting the study or relevant analyses were not performed [15].

#### Evidence synthesis

Finally, a qualitative synthesis of the evidence per measurement property, per PROM was constructed to come to an overall conclusion of PROM quality. If consistent (i.e., ≥ 75% of the results are either rated ‘sufficient’ or ‘insufficient’), the results of the individual studies on measurement properties were qualitatively summarized and again rated against the criteria for good measurement properties. If inconsistent, an explanation for this inconsistency was sought. When the inconsistency remained unexplained, the overall result was rated as ‘inconsistent’ (±). An ‘indeterminate’ (?) rating was given when the individual results were all rated as ‘indeterminate’ [15].

After qualitatively synthesizing and rating the overall results per measurement property, per PROM, the quality of this evidence was graded. In accordance with COSMIN guidelines, a modified Grading of Recommendations Assessment, Development, and Evaluation (GRADE) approach was used for grading the evidence [15]. The summarized results were graded as ‘high’, ‘moderate’, ‘low’ or ‘very low’, based on three factors: risk of bias (based on methodological quality), inconsistency and imprecision (i.e. sample size). The fourth factor ‘indirectness’ was not taken into consideration in evaluating evidence quality, this review only included studies with a predefined and fixed patient population. If the quality of the summarized result was rated ‘inconsistent’ or ‘indeterminate’, the quality of the evidence could not be graded [15].

The above-mentioned subsequent steps of the COSMIN evaluation were performed by two reviewers (JPR and TFF) independently. If consensus could not be reached during any of the evaluation procedures, an additional reviewer (IN and/or CJA) was consulted. For evaluating inter-rater agreement, a percentage agreement was calculated by dividing the number of ratings which the reviewers agreed on, by the total number of ratings given by the two reviewers. In accordance with the criterium for assessing inter-rater agreement proposed by Mokkink et al. [22], the inter-rater agreement of the reviewers was considered appropriate when reviewers reached > 80% agreement.

## Results

**Fig. 1:**
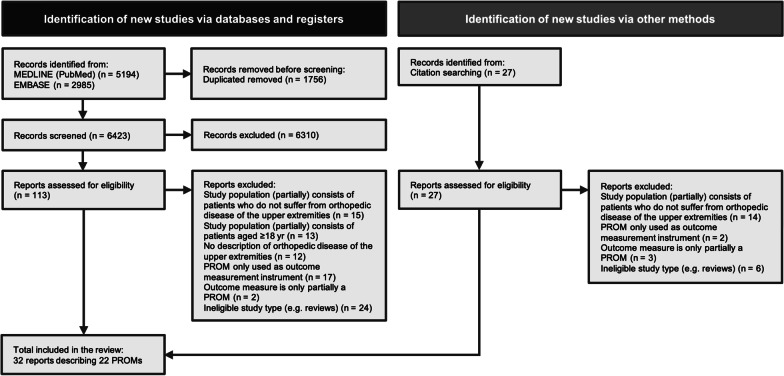
PRISMA flowchart

The literature search initially identified 8179 articles. After duplicates were removed, 6423 articles remained. Of these 6423 references, 113 were deemed eligible for inclusion after screening the titles and abstracts. As a result of hand-searching the bibliographies of these eligible articles, 27 potentially relevant citations were identified. The full-text assessment of the remaining 140 articles resulted in the inclusion of 32 original reports. The PRISMA flow diagram describing the selection process is shown in Fig. 1.

The inter-rater agreement (percentage agreement) was calculated to be 94% and therefore considered appropriate.

### General characteristics of included studies and instruments

Table 1 details the key characteristics of the articles included. In total, 32 articles reported evidence on 97 measurement properties of 22 PROMs (i.e., 15 original English PROMs and 7 cultural adaptations). The measurement property most frequently evaluated was construct validity, with 25 articles reporting on at least one construct validity assessment (e.g., hypotheses testing for construct validity). In contrast, responsiveness was evaluated in only four articles [23–26].

**Table 1:** Characteristics of the included studies

PROM	References	*n*	Age Mean (SD, range) yr	Gender % female	Disease	Country	Language
ABILHAND-Kids (Original version)	[41]	20	7.6 (2.4, 4–12)	25%	RD	The Netherlands	English
	[40]	20	8.7 (2.9, 4–12)	50%	ULRD	The Netherlands	English
	[42]	27	10 (4)	41%	Unilateral or bilateral CP	The Netherlands	English
	[48]	16	13 (2.3, 9–17)	56%	Spastic, unilateral CP	Germany	German (translational process not documented)
	[33]	113	10 (6–15)	41%	CP	Belgium	French
	[23]	52	9.1 (1.9, 6–12)		Unilateral, spastic CP	USA, The Netherlands, Belgium	English
ABILHAND-Kids (Ukrainian version)	[27]	113	10.3 (2.9, 6–16)	40%	CP	Ukraine	Ukrainian
ABILHAND-Kids (Danish version)	[28]	150	10 (2.7, 6–15)	40.7%	CP	Denmark	Danish
ABILHAND-Kids (Turkish version)	[29]	109	9.3 (2.9, 6–15)	43%	CP	Turkey	Turkish
ABILHAND-Kids (Arabic version)	[30]	154	7.4 (2.9)	45.5%	CP	Saudi Arabia	Arabic
ABILHAND-Kids (Persian version)	[31]	50	7.9 (2.2, 6–15)	40%	CP	Iran	Persian
ChARM	[36]	148	10.1 (3.3, 4.7–16.9)	39%	CP	UK	English
CHEQ	[49]	34	12.1 (3.9)	47%	Unilateral CP	Sweden	Swedish (translational process not documented)
	[37]	242	9.8 (3.4)	43%	Unilateral CP	Australia, UK, Israel, Italy, the Netherlands, Sweden	English, Hebrew, Italian, Dutch, Swedish (translational process not documented)
	[34]	86	12 (3)	51%	Unilateral CP, OBPP, ULRD	Sweden	English
CHQ	[50]	18	11.6 (10–17)	72%	NBPP	USA	English
CHSQ (Original version)	[38]	123	7.17 (2.57)	28.5%	Various known disabilities (e.g., cerebral palsy, brachial plexus birth palsy)	Australia, Taiwan	English, Taiwan Chinese
CHSQ (Turkish version)	[32]	112	7.39 (2.51, 3–12)	39%	Hemiplegic CP	Turkey	Turkish
DHI	[43]	23	10.87 (2.8, 7–16)	39.2%	Unilateral CP	Turkey	English
HUH	[44]	260	NBPP group: median age 6.9 (3.0–10.5) UCP group: median age 6.4 (3.0–10.8)	NBPP: 52% UCP: 49%	NBPP or unilateral CP	The Netherlands	English
	[35]	322	Unilateral CP group: 6.5 (2.2, 3.0–10.8) NBPP group: 6.8 (2.0, 3.0–10.4)	Unilateral CP: 52% NBPP: 50%	Unilateral CP, NBPP	The Netherlands	English
IMAL	[51]	66	1.14 (0.44)	52%	Hemiplegic/quadriplegic CP	USA	English
PEDI self-care domain	[52]	45	5.1 (3.6–6.8)	64%	OBPP	Canada	English
PODCI	[53]	23	5.6 (3.5–8.6)	61%	BPBP	USA	English
	[54]	150	5 (2–10)	55%	BPBP	USA	English
	[24]	23	6.3 (4.4–12.8)	70%	BPBP	USA	English
	[50]	18	11.6 (10–17)	72%	NBPP	USA	English
	[55]	109	- (-)	46%	Congenital upper limb differences	USA	English
PODCI (v2.0; Original version)	[25]	125	11 (2–18)	43.2%	Acute hand and wrist injuries	USA	English
PODCI (v2.0; Dutch version)	[26]	10	5.3 (2.4)	50%	NBPP	The Netherlands	Dutch
PROMIS – Upper Extremity item bank (short form, CAT)	[56]	32	11.4 (3.9)	41%	Congenital hand differences	USA	English
QuickDASH	[57]	149	- (8–18)	48%	Several types of upper extremity injuries	USA	English
Revised PMAL	[39]	61	4.5 (-)	39%	Spastic hemiplegic CP	Australia	English

SD = standard deviation, yr = year, CP = cerebral palsy, ULDR = upper limb reduction deficiencies, RD = radius deficiencies, OBPP = obstetric brachial plexus palsy, NBPP = neonatal brachial plexus palsy, BPBP = brachial plexus birth palsy, ChARM = Children’s Arm Rehabilitation Measure, CHEQ = Children's Hand-use Experience Questionnaire, CHQ = Child Health Questionnaire, CHSQ = Children’s Hand-Skills ability Questionnaire, DHI = Duruöz Hand Index, HUH = Hand-Use-at-Home questionnaire, IMAL = Infant Motor Activity Log, PEDI = Pediatric Evaluation of Disability Inventory, PODCI = Pediatric Outcomes Data Collection Instrument, PROMIS = Patient-Reported Outcomes Measurement Information System, CAT = computer-adaptive test, DASH = Disabilities of the Arm, Shoulder and Hand, PMAL = Pediatric Motor Activity Log

In agreement with COSMIN methodology, each version of a questionnaire was considered a separate PROM (i.e., cross-cultural adapted versions or revised versions) [15]. The characteristics of the instruments included in this review are shown in Table 2. English versions of PROMs were assessed most frequently. Studies performing cross-cultural adaptation and subsequent validation were scarce. Only seven culturally adapted PROM versions were evaluated in validation studies [26–32].

**Table 2:** Characteristics of the included PROMs

PROM (reference to first article)	Construct(s)	Target population	Mode of administration	Recall period	(Sub)scale(s) (number of items)	Response options	Range of scores/scoring	Original language	Available translations*
ABILHAND-Kids [33]	Manual ability	Children with cerebral palsy (> 6 yr)	Parent/proxy-report	3 months	1 scale (21 items)	3-level Likert rating scale	0–42 (raw sum score)	French/English	Ukrainian, Danish, Turkish, Arabic, Persian
ChARM [36]	Upper limb activity limitation	Children with cerebral palsy (5-16 yr)	Parent/proxy-report		1 scale (19 items)	Individual items have a differing number of response options		English	
CHEQ [34]	Perceived problems with bimanual activities	Children with unilateral dysfunction (6-18 yr)	Parent/proxy- or self-report with assistance from parents/caregivers (for children ≤ 12 yr) Self-report (for children > 12 yr)		3 scales (29 items)	4-category rating scale		English	
CHQ [50]	Health-related quality of life	Children and adolescents (5-18 yr)	Parent/proxy-report Self-report	Varies: ‘last 4 weeks’, ‘in general’	10 physical and psychosocial concepts (not reported for this CHQ-version)	4–6 level rating scale	Scores at concept-level Summary score (parent-reported version only)	English	
CHSQ [38]	Manual ability	Children with disabilities (2-12 yr)	Parent/proxy-report	3 months	3 domains (21 items)	3-level Likert rating scale		English	Turkish
DHI [43]	Functional disability	Adults with disabilities	Self-report		1 scale that can be subdivided into 3 ‘factors’ (18 items)	5-point Likert rating scale	0–90	English	
HUH [35]	The amount of spontaneous useof the affected hand	Children with unilateralupper limb paresis (3-10 yr)	Parent/proxy-report		1 scale (18 items)	5-point rating scale	Sum score (range 0–36) or Hand-Use-at-Home score in logits (interval scale, range − 4.69–5.17)	English	
IMAL [51]	Caregiver perception of upper limb-use during daily activities	Children with neurological and functional impairments (< 2 yr)	Parent/proxy-report		2 subscales (20 items)	5-point Likert rating scale		English	
PEDI self-care domain [52]	Ability to perform self-care activities	Children with physical disabilities	Parent/proxy-report		Self-care domain (7 items)	0–100		English	
PODCI [53]	Perceived limitations	Children with musculoskeletal disorders	Parent/proxy-report Self-report		5 subscales, 1 total score (114 items)	0–100	0–100 (normalized score)	English	
PODCI (v2.0) [25]	Perceived limitations	Children with musculoskeletal disorders	Parent/proxy-report (for children 2-10 yr) Self-report (for children 11-18 yr)		5 subscales, 1 total score (83/86 items)	0–100	0–100 (standardized score)	English	Dutch
PROMIS – Upper Extremity item bank (short form) [56]	Upper extremity function	The general population and children or adults living with chronic conditions	Parent/proxy-report Self-report	7-day recall period	1 scale (8 items)	5-point Likert rating scale	0–100 (normalized T-scores)	English	
PROMIS Upper Extremity item bank (CAT) [56]	Upper extremity function	The general population and children or adults living with chronic conditions	Self-report	7-day recall period	1 scale (min 5 items, max 12 items)	5-point Likert rating scale	0–100 (normalized T-scores)	English	
QuickDASH [57]	Upper extremity function	Adult patients with disabilities of the shoulder, arm, and/or hand	Self-report		1 scale (11 items)	5-point Likert rating scale	0–100 (summative scale)	English	
Revised PMAL [39]	Upper limb-use in real-life situations	Children with cerebral palsy (6mo-8 yr)	Parent/proxy-report		2 subscales (number of items in revised PMAL unknown)	3-level Likert rating scale	0–2 per question (collapsed rating scale)	English	

Information adapted exclusively from studies included in this review

yr = year, mo = month, ChARM = Children’s Arm Rehabilitation Measure, CHEQ = Children's Hand-use Experience Questionnaire, CHQ = Child Health Questionnaire, CHSQ = Children’s Hand-Skills ability Questionnaire, DHI = Duruöz Hand Index, HUH = Hand-Use-at-Home questionnaire, IMAL = Infant Motor Activity Log, PEDI = Pediatric Evaluation of Disability Inventory, PODCI = Pediatric Outcomes Data Collection Instrument, PROMIS = Patient-Reported Outcomes Measurement Information System, CAT = computer-adaptive test, DASH = Disabilities of the Arm, Shoulder and Hand, PMAL = Pediatric Motor Activity Log

^*^PROM translations that have been cross-cultural adapted and/or validated in the population of interest of this review

### Synthesized evidence

The results of the methodological quality assessment and criteria for good measurement properties ratings of the individual studies are presented in Table 3. In Table 4, for each PROM the qualitatively summarized results per measurement property, their overall quality rating (criteria for good measurement properties) and evidence quality grade (modified GRADE approach) are detailed. The detailed results of each study on a measurement property of a PROM included in this review, can be found in Additional file 1: Appendix 2.

**Table 3:** Methodological quality and ratings of measurement properties of the included PROMs

PROM	Ref	Measurement property	Methodological quality	Rating*
ABILHAND-Kids (Original version)	Buffart et al. [41]	Reliability	Adequate	+
		Measurement error	Adequate	?
		Hypotheses testing for construct validity: convergent validity	Adequate	10-/1 +
		Hypotheses testing for construct validity: discriminative validity	Very good	
	Buffart et al. [40]	Reliability	Doubtful	+
		Measurement error	Doubtful	?
		Hypotheses testing for construct validity: convergent validity	Adequate	5 +
		Hypotheses testing for construct validity: discriminative validity	Adequate	
	De Jong et al. [42]	Reliability	Doubtful	+
		Measurement error	Doubtful	?
	Klotz et al. [48]	Hypotheses testing for construct validity: convergent validity	Doubtful	1-/1 +
	Arnould et al. [33]	PROM development	Inadequate	
		Structural validity	Adequate	–
		Internal consistency	Very good	?
		Reliability	Doubtful	?
		Hypotheses testing for construct validity: discriminative validity	Doubtful	2 + ^§^
	Bleyenheuft et al. [23]	Responsiveness: construct approach (hypotheses testing)		
		Comparison with other outcome measurement instruments	Inadequate	?
		Comparison between subgroups	Very good	?
		Before and after intervention	Doubtful	?
ABILHAND-Kids (Ukrainian version)	Hasiuk et al. [27]	Structural validity	Adequate	–
		Internal consistency	Very good	?
		Cross-cultural validity	Doubtful	–
ABILHAND-Kids (Danish version)	Hansen et al. [28]	Structural validity	Adequate	–
		Internal consistency	Very good	?
		Measurement invariance	Adequate	–
		Reliability	Very good	+
		Measurement error	Very good	?
ABILHAND-Kids (Turkish version)	Şahin et al. [29]	Structural validity	Adequate	?
		Internal consistency	Very good	?
		Measurement invariance	Inadequate	+
		Reliability	Doubtful	+
		Hypotheses testing for construct validity: convergent validity	Very good	2 +
ABILHAND-Kids (Arabic version)	Alnahdi et al. [30]	Structural validity	Adequate	–
		Internal consistency	Very good	?
		Measurement invariance	Inadequate	+
		Reliability	Inadequate	+
		Measurement error	Inadequate	?
		Hypotheses testing for construct validity: convergent validity	Adequate	1-/6 +
ABILHAND-Kids (Persian version)	Mohammadkhani-Pordanjani et al. [31]	Structural validity	Doubtful	+
		Internal consistency	Very good	+
		Cross-cultural validity	Inadequate	–
		Measurement invariance	Inadequate	+
		Reliability	Inadequate	+
		Measurement error	Inadequate	?
		Hypotheses testing for construct validity: discriminative validity	Doubtful	1 +
ChARM	Preston et al. [36]	PROM development	Inadequate	
		Structural validity	Adequate	–
		Internal consistency	Very good	?
		Hypotheses testing for construct validity: discriminative validity	Doubtful	1 +
CHEQ	Ryll et al. [49]	Hypotheses testing for construct validity: convergent validity	Adequate	2 +
	Amer et al. [37]	Structural validity	Adequate	?
		Internal consistency	Very good	?
		Reliability	Doubtful	+
	Sköld et al. [34]	PROM development	Doubtful	
		Structural validity	Doubtful	?
		Internal consistency	Very good	?
CHQ	Squitieri et al. [50]	Hypotheses testing for construct validity: discriminative validity	Inadequate	?
CHSQ (Original version)	Chien et al. [38]	Structural validity	Adequate	?
		Internal consistency	Very good	?
		Cross-cultural validity	Inadequate	–
		Hypotheses testing for construct validity: convergent validity	Adequate	2-/5 +
CHSQ (Turkish version)	Gün et al. [32]	Internal consistency	Very good	?
		Reliability	Doubtful	+
		Hypotheses testing for construct validity: convergent validity	Adequate	1 +
DHI	Sanal-Top et al. [43]	Internal consistency	Very good	?
		Reliability	Inadequate	+
		Hypotheses testing for construct validity: convergent validity	Adequate	?
HUH	Van der Holst et al. [44]	Reliability	Doubtful	+
		Measurement error	Doubtful	?
		Hypotheses testing for construct validity: convergent validity	Very good	5 +
		Hypotheses testing for construct validity: discriminative validity	Very good	
	Geerdink et al. [35]	PROM development	Doubtful	
		Structural validity	Adequate	?
		Internal consistency	Very good	?
		Hypotheses testing for construct validity: discriminative validity	Doubtful	2 +
IMAL	Carey et al. [51]	Internal consistency	Very good	?
		Reliability	Doubtful	?
		Measurement error	Doubtful	?
		Hypotheses testing for construct validity: convergent validity	Adequate	?
		Hypotheses testing for construct validity: discriminative validity	Adequate	
PEDI self-care domain	Ho et al. [52]	Hypotheses testing for construct validity: discriminative validity		
		OBPP versus peers	Doubtful	1-/1 +
		OBPP with hand impairment versus OBPP without hand impairment	Adequate	
PODCI	Huffman et al. [53]	Hypotheses testing for construct validity: discriminative validity	Doubtful	5 +
	Bae et al. [54]	Hypotheses testing for construct validity: convergent validity	Doubtful	?/6 +
		Hypotheses testing for construct validity: discriminative validity	Doubtful	
	Dedini et al. [24]	Responsiveness: construct approach		
		Before and after intervention	Inadequate	2-/4 +
	Squitieri et al. [50]	Hypotheses testing for construct validity: discriminative validity	Inadequate	?
	Wall et al. [55]	Hypotheses testing for construct validity: discriminative validity	Inadequate	?
PODCI (v2.0; Original version)	Kunkel et al. [25]	Internal consistency	Very good	?
		Hypotheses testing for construct validity: discriminative validity	Doubtful	?
		Responsiveness: construct approach		
		Before and after intervention	Inadequate	?
PODCI (v2.0; Dutch version)	Van der Holst et al. [26]	Internal consistency	Very good	?
		Reliability	Inadequate	–
		Hypotheses testing for construct validity: convergent validity	Adequate	2 +
		Responsiveness: construct approach		
		Before and after intervention	Inadequate	?
PROMIS – Upper Extremity item bank (short form)	Waljee et al. [56]	Hypotheses testing for construct validity: convergent validity	Adequate	3 +
PROMIS – Upper Extremity item bank (CAT)	Waljee et al. [56]	Hypotheses testing for construct validity: convergent validity	Adequate	3 +
QuickDASH	Quatman-Yates et al. [57]	Internal consistency	Very good	?
		Hypotheses testing for construct validity: convergent validity	Doubtful	1 +
Revised PMAL	Wallen et al. [39]	Structural validity	Doubtful	?
		Internal consistency	Very good	?
		Reliability	Doubtful	+
		Hypotheses testing for construct validity: discriminative validity	Doubtful	2 +

ChARM = Children’s Arm Rehabilitation Measure, CHEQ = Children's Hand-use Experience Questionnaire, CHQ = Child Health Questionnaire, CHSQ = Children’s Hand-Skills ability Questionnaire, DHI = Duruöz Hand Index, HUH = Hand-Use-at-Home questionnaire, IMAL = Infant Motor Activity Log, PEDI = Pediatric Evaluation of Disability Inventory, PODCI = Pediatric Outcomes Data Collection Instrument, PROMIS = Patient-Reported Outcomes Measurement Information System, CAT = computer-adaptive test, DASH = Disabilities of the Arm, Shoulder and Hand, PMAL = Pediatric Motor Activity Log

^*^The result of each study on a measurement property of a PROM was rated against the updated criteria for good measurement properties: – = insufficient; +  = sufficient; ? = indeterminate

^§^Number of hypotheses tested (2) and if the hypotheses were confirmed ( +) or rejected (-) in the study

**Table 4:** Synthesized evidence

PROM (refs)	Measurement property	Summarized result	Overall rating*	Quality of evidence^§^
ABILHAND-Kids (Original version) [23, 33, 40–42, 48]	Structural validity	INFIT mean square range 0.66–1.18; OUTFIT mean square range 0.45–1.55	–	Moderate
	Internal consistency	Person separation reliability coefficient 0.94	?	
	Reliability	ICC range = 0.81–0.91	+	Moderate
	Measurement error	SEM = 1.7; SDD_95_ = 6.7; SDD_95_/range = 0.16; SEM = 1.9; SDD_90_ = 4.8; SDD/range = 0.11; LOA = -2.06–1.40	?	
	Construct validity	9 out of 20 hypotheses confirmed	±	
	Responsiveness	RM ANOVA F = 29.89, p < 0.001; Effect size T1vsT2 = 0.916, T2vsT3 = 0.158; Correlation changes measured by PEDI and ABILHAND-Kids Spearman r = 0.430, p = 0.003; Correlation changes measured by AHA and ABILHAND-Kids Pearson r = –0.104, p = 0.493	?	
ABILHAND-Kids (Ukrainian version) [27]	Structural validity	Standardized residuals range = -2.19–1.58	–	Moderate
	Internal consistency	Person separation index = 0.95	?	
	Cross-cultural validity	3 major DIF’s were observed across countries (Ukrainian versus Belgian cohort)	–	Moderate
ABILHAND-Kids (Danish version) [28]	Structural validity	TLI = 0.98; CFI = 0.98; RMSEA = 0.07; SRMR = 0.07 Fit residuals (z) range = -2.178–2.170	–	Moderate
	Internal consistency	Cronbach’s alpha = 0.96	?	
	Measurement invariance	1 non-uniform DIF was observed across age groups	–	Moderate
	Reliability	ICC2.1 = 0.97 (95% CI 0.95–0.98)	+	High
	Measurement error	SE = 0.5; LOAs range: –4.8–5.5; SDC = 5.15 points	?	
ABILHAND-Kids (Turkish version) [29]	Structural validity	Residual (z) range = -1.636–1.934	?	
	Internal consistency	Cronbach’s alpha = 0.94	?	
	Measurement invariance	No DIF was observed	+	Very low
	Reliability	ICC = 0.98 (95% CI 0.98–1.00)	+	Very low
	Construct validity	2 out of 2 hypotheses confirmed	+	High
ABILHAND-Kids (Arabic version) [30]	Structural validity	Unidimensionality T-Tests (CI): 6.08% significant tests (lower limit of 95% CI = 2.60); Fit residual range = -2.06–2.01	–	Moderate
	Internal consistency	Person separation index = 0.93	?	
	Measurement invariance	No DIF was observed	+	Very low
	Reliability	ICC_agreement_ = 0.98 (95% CI 0.97–0.99)	+	Very low
	Measurement error	SEM_agreement_ = 0.24; MDC_95_ = 0.68	?	
	Construct validity	6 out of 7 hypotheses confirmed	+	Moderate
ABILHAND-Kids (Persian version) [31]	Structural validity	*χ*.^2^ probability = 0.40; PCA on the residuals, first residual factor accounts for 13% of the observed variance; Standardized residuals range = -1.34–1.60	+	Low
	Internal consistency	Cronbach’s alpha = 0.963	+	Moderate
	Cross-cultural validity	2 major DIF’s were observed across countries	–	Very low
	Measurement invariance	No DIF was observed	+	Very low
	Reliability	ICC_agreement_ = 0.7 (CI 95% 0.33–0.85)	+	Very low
	Measurement error	SEM for CP measure = 11.21% (1.16 logits, raw score of 2.21); SDC for CP measure = 31.07% (3.21 logits, raw score of 6.13)	?	
	Construct validity	1 out of 1 hypothesis confirmed	+	Very low
ChARM [36]	Structural validity	Unidimensionality T-Tests (CI): 8% significant tests, lower limit of 95% CI = 4.6; Fit residuals range = -1.603–1.484	–	Moderate
	Internal consistency	Cronbach’s alpha = 0.95	?	
	Construct validity	1 out of 1 hypothesis confirmed	+	Low
CHEQ [34, 37, 49]	Structural validity	Rasch analyses showed misfits (INFIT mean square > 1.5 and/or Z-standardized values < -2 or > 2) for several items of all three subscales	?	
	Internal consistency	Three CHEQ subscales: Person separation reliability coefficient range = 0.89–0.94	?	
	Reliability	Opening questions: ‘performing the activity independently’ average κ = 0.63, ‘using the affected hand as support or to grasp’ average κ = 0.57; Three CHEQ subscales: average ICC 0.87–0.91	+	Very low
	Construct validity	2 out of 2 hypotheses confirmed	+	Very low
CHQ [50]	Construct validity	No hypotheses were defined a priori	?	
CHSQ (Original version) [38]	Structural validity	‘Leisure and play domain’: INFIT mean square range = 0.8–1.5, INFIT Zstd range = -1.6–2.8; OUTFIT mean square range = 0.7–1.5, OUTFIT Zstd range = -1.7–1.8 ‘School/education domain’: INFIT mean square range = 0.7–1.2, INFIT Zstd range = -2.6–1.1; OUTFIT mean square range = 0.6–1.1, OUTFIT Zstd range = -2.1–0.4 ‘Activities of daily living domain’: INFIT mean square range = 0.7–1.2, INFIT Zstd range = -1.6–1.3; OUTFIT mean square range = 0.5–1.4, OUTFIT Zstd range = -1.4–0.8	?	
	Internal consistency	Three CHSQ domains: Person reliability coefficient range = 0.67–0.75	?	
	Cross-cultural validity	7 items with DIF by cultural difference (Australian versus Taiwanese cohort)	–	Very low
	Construct validity	5 out of 7 hypotheses confirmed	±	
CHSQ (Turkish version) [32]	Internal consistency	Three CHSQ-TR subscales: Cronbach’s alpha range = 0.83–0.86	?	
	Reliability	Three CHSQ-TR subscales; ICC range = 0.98–0.99	+	Low
	Construct validity	1 out of 1 hypothesis confirmed	+	Moderate
DHI [43]	Internal consistency	Cronbach’s alpha range = 0.83–0.94	?	
	Reliability	ICC range = 0.84–0.93	+	Very low
	Construct validity	No hypotheses were defined a priori	?	
HUH [35, 44]	Structural validity	INFIT mean square range = 0.78–1.39; OUTFIT mean square range = 0.71–1.36	?	
	Internal consistency	Cronbach’s alpha = 0.941	?	
	Reliability	ICC = 0.89 (95% IC 0.81–0.93)	+	Very low
	Measurement error	SEM (logits) = 0.599; SDC_individual_ (logits) = 1.66; SDC_group_ (logits) = 0.22	?	
	Construct validity	7 out of 7 hypotheses confirmed	+	High
IMAL [51]	Internal consistency	Two IMAL subscales: Cronbach’s alpha range = 0.94–0.95	?	
	Reliability	Two IMAL subscales: Spearman’s correlation range = 0.64–0.70	?	
	Measurement error	‘How Often’ scale: SEM = 0.66 ‘How Well scale: SEM = 0.61	?	
	Construct validity	No hypotheses were defined a priori	?	
PEDI self-care domain [52]	Construct validity	1 out of 2 hypotheses confirmed	±	
PODCI [24, 50, 53–55]	Construct validity	11 out of 11 predefined hypotheses confirmed; for several analyses hypotheses could not be defined a priori	±	
	Responsiveness	4 out of 6 hypotheses confirmed	±	
PODCI (v2.0; Original version) [25]	Internal consistency	Cronbach’s alpha range = 0.82–0.93	?	
	Construct validity	No hypotheses were defined a priori	?	
	Responsiveness	Moderate-large SRM (0.38–1.27)/effect size (0.32–1.37) for UE function, mobility, pain/comfort, happiness, global function; SRM 0.12/effect size 0.14 for sports/physical	?	
PODCI (v2.0; Dutch version) [26]	Internal consistency	Cronbach’s alpha range = 0.161–0.928	?	
	Reliability	4 subscales and total score: ICC = 0.636–0.972 (p < 0.025) ‘Pain and comfort’-subscale: ICC = 0.022 (p = 0.476)	–	Very low
	Construct validity	2 out of 2 hypotheses confirmed	+	Very low
	Responsiveness	No hypotheses were defined a priori	?	
PROMIS – Upper Extremity item bank (short form) [56]	Construct validity	3 out of 3 hypotheses confirmed	+	Very low
PROMIS – Upper Extremity item bank (CAT) [56]	Construct validity	3 out of 3 hypotheses confirmed	+	Very low
QuickDASH [57]	Internal consistency	Cronbach’s alpha = 0.91	?	
	Construct validity	Results in line with 1 hypothesis	+	Low
Revised PMAL [39]	Structural validity	‘How Often’ scale: EU associated with the first PCA contrast = 2.6 ‘How Well’ scale: EU associated with the first PCA contrast = 2.5	?	
	Internal consistency	Two rPMAL subscales: Person reliability index range = 0.89–0.90	?	
	Reliability	Two rPMAL subscales: ICC range = 0.93–0.94	+	Very low
	Construct validity	2 out of 2 hypotheses confirmed	+	Very low

ICC = intraclass correlation coefficient, SEM = standard error of measurement, SDD = smallest detectable difference, LOA = limits of agreement, DIF = differential item functioning, TLI = Tucker Lewis index, CFI = Comparative fit index, RMSEA = root mean square error of approximation, SRMR = standardized root mean square residual, MDC = minimal detectable change, SDC = smallest detectable change, PCA = Principal Component Analysis, SRM = standard response mean; ChARM = Children’s Arm Rehabilitation Measure, CHEQ = Children's Hand-use Experience Questionnaire, CHQ = Child Health Questionnaire, CHSQ = Children’s Hand-Skills ability Questionnaire, DHI = Duruöz Hand Index, HUH = Hand-Use-at-Home questionnaire, IMAL = Infant Motor Activity Log, PEDI = Pediatric Evaluation of Disability Inventory, PODCI = Pediatric Outcomes Data Collection Instrument, PROMIS = Patient-Reported Outcomes Measurement Information System, CAT = computer-adaptive test, DASH = Disabilities of the Arm, Shoulder and Hand, PMAL = Pediatric Motor Activity Log

^*^The results of the different studies on a particular measurement property of a PROM were qualitatively summarized and then rated against the updated criteria for good measurement properties: – = insufficient; +  = sufficient; ±  = inconsistent; ? = indeterminate

^§^The quality of the evidence was graded by using a modified GRADE approach

#### Content validity

No studies evaluating the content validity of a PROM were considered eligible for inclusion in this review. Therefore, only the methodological quality of the included PROM development studies was determined. As each of the included development studies did not report on a pilot study assessing the comprehensibility and comprehensiveness of the instrument, the overall methodological quality of the four PROM development studies was rated as ‘inadequate’ or ‘doubtful’ [33–36].

#### Structural validity

Structural validity was evaluated for eleven of the included PROMs [27–39]. Five studies assessed the structural validity of a cultural adaptation of the ABILHAND-Kids questionnaire [27–31]. Only one PROM demonstrated evidence for sufficient structural validity: the Persian adaptation of the ABILHAND-Kids questionnaire [31]. For the other PROMs, the results of the structural validity analyses did not meet the COSMIN criteria for good measurement properties (mostly regarding the range of goodness-of-fit statistics) [27, 28, 30, 33, 36], the authors failed to report on important aspects of the IRT/Rasch analyses [29, 35, 38] and/or the subscales were only separately evaluated, which does not provide evidence for structural validity of the instrument as a whole [34, 37–39].

#### Internal consistency

For internal consistency analyses to be interpreted correctly, an instrument should at least show low-quality evidence for sufficient structural validity [15]. Therefore, only the internal consistency analysis of the Persian version of the ABILHAND-Kids questionnaire was rated [31]. For the other PROMs, the results of the internal consistency analyses were reported and an ‘indeterminate’ rating was given.

#### Other measurement properties

Thirteen of the included PROMs demonstrated evidence for sufficient test–retest reliability [26, 28–32, 37, 39–44]. Only the Dutch version of the Pediatric Outcomes Data Collection Instrument (PODCI) demonstrated evidence for insufficient reliability with ICC values ranging from 0.022–0.972 for the different subscales [26].

The results of analyses on measurement error were all rated as ‘indeterminate’, since information on minimal important change (MIC) had not yet been published for the PROMs included in this review.

## Discussion

This study is the first systematic review to provide a comprehensive overview of evidence on the psychometric properties of PROMs used for evaluating children with impairment of the upper extremity. Twenty-two PROMs, measuring various constructs, were included and evaluated using the updated version of the extensive COSMIN methodology to ensure a high-quality assessment. Additionally, this study provides an opportunity to formulate evidence-based recommendations for PROM-selection and increase awareness on proper PROM utilization in clinical practice and research.

When basing recommendations for PROM-selection exclusively on the quality of their measurement properties, the current lack of evidence on PROM-quality has the consequence that the 22 pediatric orthopedic PROMs included in this review have the potential to be recommended for use, but further research is required to assess their quality. Evidence on content validity and internal consistency of a PROM is fundamental to formulating a transparent, evidence-based recommendation [15]. However, content validity, which can be considered the most important psychometric property of a PROM [21], was not evaluated for any of the included PROMs. Internal consistency was evaluated for 16 of the 22 pediatric orthopedic PROMs. Unfortunately, only one study provided sufficient evidence to rate the internal consistency of the questionnaire (ABILHAND-Kids: Persian version). All other studies provided insufficient evidence on structural validity, which is essential for correctly interpreting the results of internal consistency analyses [15]. Furthermore, psychometric properties of only four of the questionnaires were validated in more than one validation study (ABILHAND-Kids (original version), PODCI, Children's Hand-use Experience Questionnaire and Hand-Use-at-Home questionnaire). Even though these instruments were evaluated most frequently, the quality of two thirds of their measurement properties was rated as ‘indeterminate’ or ‘inconsistent’, with the PODCI solely demonstrating inconsistent evidence. This trend was also observed for the other PROMs included in this review. Moreover, the overall quality of the included validation studies varied considerably, mainly due to insufficient sample size and/or poor methodological quality.

When exploring additional means to provide clinicians and researchers with a basis to guide their PROM-selection, formulating recommendations based on feasibility aspects of PROMs constitutes a valuable alternative approach. The term ‘feasibility’ refers to the ease with which the instrument is applied in its intended context of use and includes PROM characteristics such as completion time and length of the questionnaire [15]. Although feasibility is not considered a measurement property as it does not pertain to the quality of a PROM, feasibility aspects profoundly influence the practical utility of a PROM, especially factors influencing response rate and patient compliance such as questionnaire length [45]. The data collection method of computer-adaptive testing (CAT) uses item-response theory to minimize questionnaire length and completion time; consequently, optimizing response rates [45]. Whereas the majority of the included PROMs use traditional data collection methods, one PROM was assessed using computer-adaptive testing: the PROMIS – Upper Extremity item bank computer-adaptive test (CAT). Therefore, based on the evidence currently available, the PROMIS – Upper Extremity item bank CAT can be considered the most appropriate PROM for evaluating upper extremity function in children, when adopting this feasibility-driven approach to guiding PROM-selection.

The overall methodological quality of the four PROM development studies included in this review was rated as ‘inadequate’ or ‘doubtful’ [33–36]. For each of the instruments, the developmental process lacked a cognitive interview study or other pilot test evaluating their comprehensibility and comprehensiveness. During the development of PROMs in pediatric research, researchers must take developmental influences such as age-dependent disease-awareness and cognitive–linguistic ability, into careful consideration [46, 47]. These considerations unique to pediatric qualitative research, make developing pediatric PROMs with a high methodological quality, a strenuous and time-consuming practice. However, to ensure the questionnaire matches the perspective and needs of the patients it has been designed for, it is imperative to adequately evaluate aspects such as comprehensibility, especially for pediatric PROMs. To guarantee future pediatric orthopedic PROMs will adequately reflect the patients’ perspective on their health condition, it is vital to incorporate pilot studies assessing relevance, comprehensiveness, and comprehensibility into the development of these instruments.

Whilst conducting this systematic review, we followed the extensive and newly updated COSMIN methodology for systematic reviews of PROMs, which can be considered one of the strengths of this study. Using the COSMIN checklists sometimes requires a subjective judgement by the reviewer (e.g., in determining which measurement properties were assessed when the terms used in the article did not match the COSMIN taxonomy). This potential source of bias was addressed by two reviewers independently extracting and evaluating data and by building consensus, further strengthening the approach utilized in this review.

This review has some limitations. Even though using the COSMIN methodology guarantees a standardized and thorough approach for evaluating the included studies on measurement properties, “the worst score counts” principle applied in rating these studies can be considered reductive. As the worst rating in a COSMIN box will determine the overall result of the quality assessment, the absence of reporting on a particular evaluation step or statistical method can result in the study being rated as ‘doubtful’ or even ‘inadequate’. Consequently, a cogent argument can be made that using this principle results in the undervaluation of the already small amount of evidence available on pediatric orthopedic PROMs.

In an effort to provide a comprehensive overview of the pediatric orthopedic PROMs available to clinicians and researchers, we purposefully used broad inclusion criteria with respect to study population (e.g., any orthopedic condition in the upper extremity region) and type of instrument (e.g., self-completed as well as proxy-completed questionnaires). Subdividing the population of interest based on affected limb, body region or disease type, was limited by the paucity of evidence available on pediatric orthopedic PROMs. In addressing the challenges these broad inclusion criteria posed to the feasibility of our review, some concessions had to be made regarding the scope of our search. Consequently, only MEDLINE and EMBASE were searched omitting potentially relevant databases like CINAHL, and the timeframe was condensed, possibly preventing the inclusion of additional relevant articles.

## Conclusions

In conclusion, a comprehensive overview was given of PROMs used in pediatric orthopedic research of the upper extremity. None of the PROMs included in this review demonstrated sufficient evidence on their measurement properties to strongly recommend the use of any of these instruments in children with impairment of the upper extremity. The absence of studies on content validity for any of the included PROMs is especially worrisome, as this implies it is currently unknown if the questionnaires used in pediatric orthopedic research and clinical practice adequately reflect the construct they intend to measure. When an alternative, feasibility-driven approach to guiding PROM-selection is adopted, the PROMIS – Upper Extremity CAT can cautiously be considered the most appropriate PROM for measuring upper extremity function in children with impairment of the upper limb. The lack of evidence on PROM-quality uncovers a need for high-quality development and validation studies, and especially studies on content validity, for PROMs utilized in pediatric orthopedics.

## Supplementary Information

**Additional file 1.** **Appendix 1**: search strings MEDLINE (PubMed) and EMBASE; **Appendix 2**: Results and ratings of measurement properties of the included PROMs.

## Data Availability

All data analyzed during this study are included in this article and its supplementary files.
