# Supplementary material for: Quality of patient- and proxy-reported outcomes for children with impairment of the upper extremity: a systematic review using the COSMIN methodology
Source: J Patient Rep Outcomes. 2022 Jun 2;6:58. doi: 10.1186/s41687-022-00469-4 (PMC9163282; doi:10.1186/s41687-022-00469-4)
Supplement: Supplementary file 1 — Additional file 1. Appendix 1: search strings MEDLINE (PubMed) and EMBASE; Appendix 2: Results and ratings of measurement properties of the included PROMs. [file 41687_2022_469_MOESM1_ESM.docx]

# Appendix 1: Search strings MEDLINE (PubMed) and EMBASE

## MEDLINE (PubMed)

((((("Upper Extremity"[Mesh] OR "upper extremity"[Title/Abstract] OR "upper extremities"[Title/Abstract] OR "upper limb"[Title/Abstract] OR "upper limbs"[Title/Abstract] OR "arm"[Title] OR "arms"[Title] OR "forearm"[Title/Abstract] OR "fore-arm"[Title/Abstract] OR "shoulder*"[Title/Abstract] OR "elbow*"[Title/Abstract] OR "wrist*"[Title/Abstract] OR "hand"[Title/Abstract] OR "hands"[Title/Abstract] OR "finger*"[Title/Abstract] OR "humerus"[Title/Abstract] OR "humeral"[Title/Abstract] OR "olecranon"[Title/Abstract] OR "radius"[Title/Abstract] OR "radial"[Title/Abstract] OR "ulna"[Title/Abstract] OR "ulnar"[Title/Abstract]) NOT ("Arthritis"[Mesh])) AND (infan*[Title/Abstract] OR newborn*[Title/Abstract] OR new-born*[Title/Abstract] OR perinat*[Title/Abstract] OR neonat*[Title/Abstract] OR baby[Title/Abstract] OR baby*[Title/Abstract] OR babies[Title/Abstract] OR toddler*[Title/Abstract] OR minors*[Title/Abstract] OR boy[Title/Abstract] OR boys[Title/Abstract] OR boyfriend[Title/Abstract] OR boyhood[Title/Abstract] OR girl*[Title/Abstract] OR kid[Title/Abstract] OR kids[Title/Abstract] OR child[Title/Abstract] OR child*[Title/Abstract] OR children*[Title/Abstract] OR schoolchild*[Title/Abstract] OR school child*[Title/Abstract] OR adolescen*[Title/Abstract] OR juvenil*[Title/Abstract] OR youth*[Title/Abstract] OR teen*[Title/Abstract] OR under*age*[Title/Abstract] OR pubescen*[Title/Abstract] OR "Pediatrics"[Mesh] OR pediatric*[Title/Abstract] OR paediatric*[Title/Abstract] OR peadiatric*[Title/Abstract])) AND ((HR-PRO[tiab] OR HRPRO[tiab] OR HRQL[tiab] OR HRQoL[tiab] OR QL[tiab] OR QoL[tiab] OR quality of life[tw] OR life quality[tw] OR health index*[tiab] OR health indices[tiab] OR health profile*[tiab] OR health status[tw] OR ((patient[tiab] OR self[tiab] OR child[tiab] OR parent[tiab] OR carer[tiab] OR proxy[tiab]) AND ((report[tiab] OR reported[tiab] OR reporting[tiab]) OR (rated[tiab] OR rating[tiab] OR ratings[tiab]) OR based[tiab] OR (assessed[tiab] OR assessment[tiab] OR assessments[tiab]))) OR ((disability[tiab] OR function[tiab] OR functional[tiab] OR functions[tiab] OR subjective[tiab] OR utility[tiab] OR utilities[tiab] OR wellbeing[tiab] OR well being[tiab]) AND (index[tiab] OR indices[tiab] OR instrument[tiab] OR instruments[tiab] OR measure[tiab] OR measures[tiab] OR questionnaire[tiab] OR questionnaires[tiab] OR profile[tiab] OR profiles[tiab] OR scale[tiab] OR scales[tiab] OR score[tiab] OR scores[tiab] OR status[tiab] OR survey[tiab] OR surveys[tiab]))))) AND (("instrumentation"[MeSH Subheading] OR "methods"[MeSH Subheading] OR "Validation Study"[pt] OR "Comparative Study"[pt] OR "psychometrics"[MeSH] OR psychometr*[tiab] OR clinimetr*[tw] OR clinometr*[tw] OR "Outcome Assessment, Health Care"[MeSH] OR "outcome assessment"[tiab] OR "outcome measure*"[tw] OR "observer variation"[MeSH] OR "observer variation"[tiab] OR "Health Status Indicators"[Mesh] OR "reproducibility of results"[MeSH] OR reproducib*[tiab] OR "discriminant analysis"[MeSH] OR reliab*[tiab] OR unreliab*[tiab] OR valid*[tiab] OR "coefficient of variation"[tiab] OR coefficient[tiab] OR homogeneity[tiab] OR homogeneous[tiab] OR "internal consistency"[tiab] OR (cronbach*[tiab] AND (alpha[tiab] OR alphas[tiab])) OR (item[tiab] AND (correlation*[tiab] OR selection*[tiab] OR reduction*[tiab])) OR agreement[tw] OR precision[tw] OR imprecision[tw] OR "precise values"[tw] OR test-retest[tiab] OR (test[tiab] AND retest[tiab]) OR (reliab*[tiab] AND (test[tiab] OR retest[tiab])) OR stability[tiab] OR interrater[tiab] OR inter-rater[tiab] OR intrarater[tiab] OR intra-rater[tiab] OR intertester[tiab] OR inter-tester[tiab] OR intratester[tiab] OR intra-tester[tiab] OR interobserver[tiab] OR inter-observer[tiab] OR intraobserver[tiab] OR intra-observer[tiab] OR intertechnician[tiab] OR inter-technician[tiab] OR intratechnician[tiab] OR intra-technician[tiab] OR interexaminer[tiab] OR inter-examiner[tiab] OR intraexaminer[tiab] OR intra-examiner[tiab] OR interassay[tiab] OR inter-assay[tiab] OR intraassay[tiab] OR intra-assay[tiab] OR interindividual[tiab] OR inter-individual[tiab] OR intraindividual[tiab] OR intra-individual[tiab] OR interparticipant[tiab] OR inter-participant[tiab] OR intraparticipant[tiab] OR intra-participant[tiab] OR kappa[tiab] OR kappa’s[tiab] OR kappas[tiab] OR repeatab*[tw] OR ((replicab*[tw] OR repeated[tw]) AND (measure[tw] OR measures[tw] OR findings[tw] OR result[tw] OR results[tw] OR test[tw] OR tests[tw])) OR generaliza*[tiab] OR generalisa*[tiab] OR concordance[tiab] OR (intraclass[tiab] AND correlation*[tiab]) OR discriminative[tiab] OR "known group"[tiab] OR "factor analysis"[tiab] OR "factor analyses"[tiab] OR "factor structure"[tiab] OR "factor structures"[tiab] OR dimension*[tiab] OR subscale*[tiab] OR (multitrait[tiab] AND scaling[tiab] AND (analysis[tiab] OR analyses[tiab])) OR "item discriminant"[tiab] OR "interscale correlation*"[tiab] OR error[tiab] OR errors[tiab] OR "individual variability"[tiab] OR "interval variability"[tiab] OR "rate variability"[tiab] OR (variability[tiab] AND (analysis[tiab] OR values[tiab])) OR (uncertainty[tiab] AND (measurement[tiab] OR measuring[tiab])) OR "standard error of measurement"[tiab] OR sensitiv*[tiab] OR responsive*[tiab] OR (limit[tiab] AND detection[tiab]) OR "minimal detectable concentration"[tiab] OR interpretab*[tiab] OR ((minimal[tiab] OR minimally[tiab] OR clinical[tiab] OR clinically[tiab]) AND (important[tiab] OR significant[tiab] OR detectable[tiab]) AND (change[tiab] OR difference[tiab])) OR (small*[tiab] AND (real[tiab] OR detectable[tiab]) AND (change[tiab] OR difference[tiab])) OR "meaningful change"[tiab] OR "ceiling effect"[tiab] OR "floor effect"[tiab] OR "Item response model"[tiab] OR IRT[tiab] OR Rasch[tiab] OR "Differential item functioning"[tiab] OR DIF[tiab] OR "computer adaptive testing"[tiab] OR "item bank"[tiab] OR "cross-cultural equivalence"[tiab]))) NOT (("Address"[Publication Type] OR "biography"[Publication Type] OR "case reports"[Publication Type] OR "comment"[Publication Type] OR "directory"[Publication Type] OR "editorial"[Publication Type] OR "festschrift"[Publication Type] OR "interview"[Publication Type] OR "Lecture"[Publication Type] OR "Legal Case"[Publication Type] OR "legislation"[Publication Type] OR "letter"[Publication Type] OR "news"[Publication Type] OR "newspaper article"[Publication Type] OR "patient education handout"[Publication Type] OR "Congress"[Publication Type] OR "consensus development conference"[Publication Type] OR "consensus development conference, nih"[Publication Type] OR "practice guideline"[Publication Type] OR "Randomized Controlled Trial"[Publication Type] OR "Adaptive Clinical Trial"[Publication Type] OR "Clinical Trial"[Publication Type] OR "Clinical Trial, Phase I"[Publication Type] OR "Clinical Trial, Phase II"[Publication Type] OR "Clinical Trial, Phase III"[Publication Type] OR "Clinical Trial, Phase IV"[Publication Type] OR "Clinical Trial Protocol"[Publication Type] OR "Controlled Clinical Trial"[Publication Type]) NOT ("animals"[MeSH Terms] NOT "humans"[MeSH Terms])) AND ((dutch[Filter] OR english[Filter]) AND (2000:2021[pdat]))

## EMBASE

('upper limb'/exp OR 'upper limb' OR 'upper extremity':ti,ab,kw OR 'upper extremities':ti,ab,kw OR 'upper limb':ti,ab,kw OR 'upper limbs':ti,ab,kw OR 'arm':ti OR 'arms':ti OR 'forearm':ti,ab,kw OR 'fore-arm':ti,ab,kw OR 'shoulder*':ti,ab,kw OR 'elbow*':ti,ab,kw OR 'wrist*':ti,ab,kw OR 'hand':ti,ab,kw OR 'hands':ti,ab,kw OR 'finger*':ti,ab,kw OR 'humerus':ti,ab,kw OR 'humeral':ti,ab,kw OR 'olecranon':ti,ab,kw OR 'radius':ti,ab,kw OR 'radial':ti,ab,kw OR 'ulna':ti,ab,kw OR 'ulnar':ti,ab,kw) NOT ('arthritis'/exp OR 'arthritis') AND ('infan*':ti,ab,kw OR 'newborn*':ti,ab,kw OR 'new-born*':ti,ab,kw OR 'perinat*':ti,ab,kw OR 'neonat*':ti,ab,kw OR 'baby':ti,ab,kw OR 'baby*':ti,ab,kw OR 'babies':ti,ab,kw OR 'toddler*':ti,ab,kw OR 'minors*':ti,ab,kw OR 'boy':ti,ab,kw OR 'boys':ti,ab,kw OR 'boyfriend':ti,ab,kw OR 'boyhood':ti,ab,kw OR 'girl*':ti,ab,kw OR 'kid':ti,ab,kw OR 'kids':ti,ab,kw OR 'child':ti,ab,kw OR 'child*':ti,ab,kw OR 'children*':ti,ab,kw OR 'schoolchild*':ti,ab,kw OR 'school child*':ti,ab,kw OR 'adolescen*':ti,ab,kw OR 'juvenil*':ti,ab,kw OR 'youth*':ti,ab,kw OR 'teen*':ti,ab,kw OR 'under*age*':ti,ab,kw OR 'pubescen*':ti,ab,kw OR 'pediatrics'/exp OR 'pediatrics' OR 'pediatric*':ti,ab,kw OR 'paediatric*':ti,ab,kw OR 'peadiatric*':ti,ab,kw) AND ('hr-pro':ti,ab,kw OR 'hrpro':ti,ab,kw OR 'hrql':ti,ab,kw OR 'hrqol':ti,ab,kw OR 'ql':ti,ab,kw OR 'qol':ti,ab,kw OR 'quality of life':ti,ab,kw OR 'life quality':ti,ab,kw OR 'health index*':ti,ab,kw OR 'health indices':ti,ab,kw OR 'health profile*':ti,ab,kw OR 'health status':ti,ab,kw OR (('patient':ti,ab,kw OR 'self':ti,ab,kw OR 'child':ti,ab,kw OR 'parent':ti,ab,kw OR 'carer':ti,ab,kw OR 'proxy':ti,ab,kw) AND ('report':ti,ab,kw OR 'reported':ti,ab,kw OR 'reporting':ti,ab,kw OR 'rated':ti,ab,kw OR 'rating':ti,ab,kw OR 'ratings':ti,ab,kw OR 'based':ti,ab,kw OR 'assessed':ti,ab,kw OR 'assessment':ti,ab,kw OR 'assessments':ti,ab,kw)) OR (('disability':ti,ab,kw OR 'function':ti,ab,kw OR 'functional':ti,ab,kw OR 'functions':ti,ab,kw OR 'subjective':ti,ab,kw OR 'utility':ti,ab,kw OR 'utilities':ti,ab,kw OR 'wellbeing':ti,ab,kw OR 'well being':ti,ab,kw) AND ('index':ti,ab,kw OR 'indices':ti,ab,kw OR 'instrument':ti,ab,kw OR 'instruments':ti,ab,kw OR 'measure':ti,ab,kw OR 'measures':ti,ab,kw OR 'questionnaire':ti,ab,kw OR 'questionnaires':ti,ab,kw OR 'profile':ti,ab,kw OR 'profiles':ti,ab,kw OR 'scale':ti,ab,kw OR 'scales':ti,ab,kw OR 'score':ti,ab,kw OR 'scores':ti,ab,kw OR 'status':ti,ab,kw OR 'survey':ti,ab,kw OR 'surveys':ti,ab,kw))) AND ('intermethod comparison'/exp OR 'intermethod comparison' OR 'data collection method'/exp OR 'data collection method' OR 'validation study'/exp OR 'validation study' OR 'feasibility study'/exp OR 'feasibility study' OR 'pilot study'/exp OR 'pilot study' OR 'psychometry'/exp OR 'psychometry' OR 'reproducibility'/exp OR 'reproducibility' OR reproducib*:ab,ti OR 'audit':ab,ti OR psychometr*:ab,ti OR clinimetr*:ab,ti OR clinometr*:ab,ti OR 'observer variation'/exp OR 'observer variation' OR 'observer variation':ab,ti OR 'discriminant analysis'/exp OR 'discriminant analysis' OR 'validity'/exp OR 'validity' OR reliab*:ab,ti OR valid*:ab,ti OR 'coefficient':ab,ti OR 'internal consistency':ab,ti OR (cronbach*:ab,ti AND ('alpha':ab,ti OR 'alphas':ab,ti)) OR 'item correlation':ab,ti OR 'item correlations':ab,ti OR 'item selection':ab,ti OR 'item selections':ab,ti OR 'item reduction':ab,ti OR 'item reductions':ab,ti OR 'agreement':ab,ti OR 'precision':ab,ti OR 'imprecision':ab,ti OR 'precise values':ab,ti OR 'test-retest':ab,ti OR ('test':ab,ti AND 'retest':ab,ti) OR (reliab*:ab,ti AND ('test':ab,ti OR 'retest':ab,ti)) OR 'stability':ab,ti OR 'interrater':ab,ti OR 'inter-rater':ab,ti OR 'intrarater':ab,ti OR 'intra-rater':ab,ti OR 'intertester':ab,ti OR 'inter-tester':ab,ti OR 'intratester':ab,ti OR 'interobeserver':ab,ti OR 'inter-observer':ab,ti OR 'intraobserver':ab,ti OR 'intertechnician':ab,ti OR 'inter-technician':ab,ti OR 'intratechnician':ab,ti OR 'interexaminer':ab,ti OR 'inter-examiner':ab,ti OR 'intraexaminer':ab,ti OR 'interassay':ab,ti OR 'inter-assay':ab,ti OR 'intraassay':ab,ti OR 'intra-assay':ab,ti OR 'interindividual':ab,ti OR 'inter-individual':ab,ti OR 'intraindividual':ab,ti OR 'intra-individual':ab,ti OR 'interparticipant':ab,ti OR 'inter-participant':ab,ti OR 'intraparticipant':ab,ti OR 'kappa':ab,ti OR 'kappas':ab,ti OR 'coefficient of variation':ab,ti OR repeatab*:ab,ti OR ((replicab*:ab,ti OR 'repeated':ab,ti) AND ('measure':ab,ti OR 'measures':ab,ti OR 'findings':ab,ti OR 'result':ab,ti OR 'results':ab,ti OR 'test':ab,ti OR 'tests':ab,ti)) OR generaliza*:ab,ti OR generalisa*:ab,ti OR 'concordance':ab,ti OR ('intraclass':ab,ti AND correlation*:ab,ti) OR 'discriminative':ab,ti OR 'known group':ab,ti OR 'factor analysis':ab,ti OR 'factor analyses':ab,ti OR 'factor structure':ab,ti OR 'factor structures':ab,ti OR 'dimensionality':ab,ti OR subscale*:ab,ti OR 'multitrait scaling analysis':ab,ti OR 'multitrait scaling analyses':ab,ti OR 'item discriminant':ab,ti OR 'interscale correlation':ab,ti OR 'interscale correlations':ab,ti OR (('error':ab,ti OR 'errors':ab,ti) AND (measure*:ab,ti OR correlat*:ab,ti OR evaluat*:ab,ti OR 'accuracy':ab,ti OR 'accurate':ab,ti OR 'precision':ab,ti OR 'mean':ab,ti)) OR 'individual variability':ab,ti OR 'interval variability':ab,ti OR 'rate variability':ab,ti OR 'variability analysis':ab,ti OR ('uncertainty':ab,ti AND ('measurement':ab,ti OR 'measuring':ab,ti)) OR 'standard error of measurement':ab,ti OR sensitiv*:ab,ti OR responsive*:ab,ti OR ('limit':ab,ti AND 'detection':ab,ti) OR 'minimal detectable concentration':ab,ti OR interpretab*:ab,ti OR (small*:ab,ti AND ('real':ab,ti OR 'detectable':ab,ti) AND ('change':ab,ti OR 'difference':ab,ti)) OR 'meaningful change':ab,ti OR 'minimal important change':ab,ti OR 'minimal important difference':ab,ti OR 'minimally important change':ab,ti OR 'minimally important difference':ab,ti OR 'minimal detectable change':ab,ti OR 'minimal detectable difference':ab,ti OR 'minimally detectable change':ab,ti OR 'minimally detectable difference':ab,ti OR 'minimal real change':ab,ti OR 'minimal real difference':ab,ti OR 'minimally real change':ab,ti OR 'minimally real difference':ab,ti OR 'ceiling effect':ab,ti OR 'floor effect':ab,ti OR 'item response model':ab,ti OR 'irt':ab,ti OR 'rasch':ab,ti OR 'differential item functioning':ab,ti OR 'dif':ab,ti OR 'computer adaptive testing':ab,ti OR 'item bank':ab,ti OR 'cross-cultural equivalence':ab,ti) NOT ('case report':de OR 'clinical trial':de OR 'controlled clinical trial':de OR 'phase 1 clinical trial':de OR 'phase 2 clinical trial':de OR 'phase 3 clinical trial':de OR 'phase 4 clinical trial':de OR 'randomized controlled trial':de) AND ([article]/lim OR [article in press]/lim OR [data papers]/lim OR [erratum]/lim OR [review]/lim OR [short survey]/lim) AND [humans]/lim AND ([dutch]/lim OR [english]/lim) AND [2000-2021]/py AND [embase]/lim

## Appendix 2: Results and ratings of measurement properties of the included PROMs

| **Ref** | **Measurement property** | **Result** | **Rating*** |
| --- | --- | --- | --- |
| **ABILHAND-Kids (Original version)** | | | |
| Buffart et al.(41) | *Reliability* | ICC = 0.91 (95% CI 0.69-0.98) | + |
|  | *Measurement error* | SEM = 1.9; SDD_90_ = 4.8; SDD/range = 0.11 | ? |
|  | *Hypotheses testing for construct validity: convergent validity and discriminative validity* | Results not in line with 10 hypotheses | 10-/1+ |
|  |  | Results in line with 1 hypothesis |  |
|  |  |  |  |
| Buffart et al.(40) | *Reliability* | ICC = 0.89 (95% CI 0.66-0.94) | + |
|  | *Measurement error* | SEM = 1.7; SDD_95_ = 6.7; SDD_95_/range = 0.16 | ? |
|  | *Hypotheses testing for construct validity: convergent validity and discriminative validity* | Results in line with 5 hypotheses | 5+ |
|  |  |  |  |
| De Jong et al.(42) | *Reliability* | ICC = 0.81 (95% CI 0.77-0.95); Repeatability coefficient = 1.82 | + |
|  | *Measurement error* | LOA = -2.06-1.40 | ? |
|  |  |  |  |
| Klotz et al.(48) | *Hypotheses testing for construct validity: convergent validity* | Results not in line with 1 hypothesis  Results in line with 1 hypothesis | 1-/1+ |
|  |  |  |  |
| Arnould et al.(33) | *Structural validity* | INFIT mean square range = 0.66-1.18; OUTFIT mean square range = 0.45-1.55 | – |
|  | *Internal consistency* | Person separation reliability coefficient = 0.94 | ? |
|  | *Reliability* | Pearson correlation coefficient R = 0.91, p <0.001 | ? |
|  | *Hypotheses testing for construct validity: discriminative validity* | Results in line with 2 hypotheses | 2+^§^ |
|  |  | | |
| Bleyenheuft et al.(23) | *Responsiveness: construct approach* | RM ANOVA F = 29.89, p<0.001; Effect size T1vsT2 = 0.916, T2vsT3 = 0.158;  Correlation changes measured by PEDI and ABILHAND-Kids Spearman r = 0.430, p = 0.003; Correlation changes measured by AHA and ABILHAND-Kids Pearson, r = –0.104, p = 0.493 | ? |
|  | **ABILHAND-Kids (Ukrainian version)** | | |
| Hasiuk et al.(27) | *Structural validity* | Standardized residuals range = -2.19-1.58 | – |
|  | *Internal consistency* | Person separation index = 0.95 | ? |
|  | *Cross-cultural validity* | 3 major DIF’s were observed across countries (Ukrainian versus Belgian cohort) | – |
|  | **ABILHAND-Kids (Danish version)** | | |
| Hansen et al.(28) | *Structural validity* | TLI = 0.98; CFI = 0.98; RMSEA = 0.07; SRMR = 0.07  Fit residuals (z) range = -2.178-2.170 | – |
|  | *Internal consistency* | Cronbach’s alpha = 0.96 | ? |
|  | *Measurement invariance* | 1 non-uniform DIF was observed across age groups | – |
|  | *Reliability* | ICC2.1 = 0.97 (95% CI 0.95-0.98) | + |
|  | *Measurement error* | SE = 0.5; LOAs range: –4.8-5.5; SDC = 5.15 points | ? |
|  | **ABILHAND-Kids (Turkish version)** | | |
| Şahin et al.(29) | *Structural validity* | Residual (z) range = -1.636-1.934 | ? |
|  | *Internal consistency* | Cronbach’s alpha = 0.94 | ? |
|  | *Measurement invariance* | No DIF was observed | + |
|  | *Reliability* | ICC = 0.98 (95% CI 0.98-1.00) | + |
|  | *Hypotheses testing for construct validity: convergent validity* | Results in line with 2 hypotheses | 2+ |
|  | **ABILHAND-Kids (Arabic version)** | | |
| Alnahdi et al.(30) | *Structural validity* | Unidimensionality T-Tests (CI): 6.08% significant tests (lower limit of 95% CI = 2.60);  Fit residual range = -2.06-2.01 | – |
|  | *Internal consistency* | Person separation index = 0.93 | ? |
|  | *Measurement invariance* | No DIF was observed | + |
|  | *Reliability* | ICC_agreement_ = 0.98 (95% CI 0.97-0.99) | + |
|  | *Measurement error* | SEM_agreement_ = 0.24; MDC_95_ = 0.68 | ? |
|  | *Hypotheses testing for construct validity: convergent validity* | Results not in line with 1 hypothesis  Results in line with 6 hypotheses | 1-/6+ |
|  | **ABILHAND-Kids (Persian version)** | | |
| Mohammadkhani-Pordanjani et al.(31) | *Structural validity* | *χ*^2^ probability = 0.40; PCA on the residuals, first residual factor accounts for 13% of the  observed variance;  Standardized residuals range = -1.34-1.60 | + |
|  | *Internal consistency* | Cronbach’s alpha = 0.963 | + |
|  | *Cross-cultural validity* | 2 major DIF’s were observed across countries | – |
|  | *Measurement invariance* | No DIF was observed | + |
|  | *Reliability* | ICC_agreement_ = 0.7 (CI 95% 0.33–0.85) | + |
|  | *Measurement error* | SEM for CP measure = 11.21% (1.16 logits, raw score of 2.21); SDC for CP measure = 31.07% (3.21 logits, raw score of 6.13) | ? |
|  | *Hypotheses testing for construct validity: discriminative validity* | Results in line with 1 hypothesis | 1+ |
|  | **ChARM** | | |
| Preston et al.(36) | *Structural validity* | Unidimensionality T-Tests (CI): 8% significant tests, lower limit of 95% CI = 4.6;  Fit residuals range = -1.603-1.484 | – |
|  | *Internal consistency* | Cronbach’s alpha = 0.95 | ? |
|  | *Hypotheses testing for construct validity: discriminative validity* | Results in line with 1 hypothesis | 1+ |
|  | **CHEQ** | | |
| Ryll et al.(49) | *Hypotheses testing for construct validity: convergent validity* | Results in line with 2 hypotheses | 2+ |
|  |  |  |  |
| Amer et al.(37) | *Structural validity* | ‘Grasp efficacy’ subscale: INFIT mean square range = 0.76-1.38  ‘Time taken’ subscale: INFIT mean square range = 0.67-1.35  ‘Feeling bothered’ subscale: INFIT mean square range = 0.73-1.70 | ? |
|  | *Internal consistency* | ‘Grasp efficacy’ subscale: Person separation reliability coefficient = 0.91  ‘Time taken’ subscale: Person separation reliability coefficient = 0.92  ‘Feeling bothered’ subscale: Person separation reliability coefficient = 0.89 | ? |
|  | *Reliability* | Opening questions: ‘performing the activity independently’ average κ = 0.63, ‘using the affected hand as support or to grasp’ average κ = 0.57; three CHEQ subscales: average ICC 0.87–0.91 | + |
|  |  |  |  |
| Sköld et al.(34) | *Structural validity* | ‘Grasp efficacy’ subscale: INFIT mean square range = 0.57-1.77  ‘Time taken’ subscale: INFIT mean square range = 0.56-1.46  ‘Feeling bothered’ subscale: INFIT mean square range = 0.55-1.39 | ? |
|  | *Internal consistency* | ‘Grasp efficacy’ subscale: Person separation reliability coefficient = 0.92  ‘Time taken’ subscale: Person separation reliability coefficient = 0.94  ‘Feeling bothered’ subscale: Person separation reliability coefficient = 0.90 | ? |
|  | **CHQ** | | |
| Squitieri et al.(50) | *Hypotheses testing for construct validity: discriminative validity* | No hypotheses were defined a priori | ? |
|  | **CHSQ (Original version)** | | |
| Chien et al.(38) | *Structural validity* | ‘Leisure and play domain’: INFIT mean square range = 0.8-1.5, INFIT Zstd range = -1.6-2.8; OUTFIT mean square range = 0.7-1.5, OUTFIT Zstd range = -1.7-1.8  ‘School/education domain’: INFIT mean square range = 0.7-1.2, INFIT Zstd range = -2.6-1.1; OUTFIT mean square range = 0.6-1.1, OUTFIT Zstd range = -2.1-0.4  ‘Activities of daily living domain’: INFIT mean square range = 0.7-1.2, INFIT Zstd range = -1.6-1.3; OUTFIT mean square range = 0.5-1.4, OUTFIT Zstd range = -1.4-0.8 | ? |
|  | *Internal consistency* | ‘Leisure and play domain’: Person reliability coefficient = 0.75  ‘School/education domain’: Person reliability coefficient = 0.75  ‘Activities of daily living domain’: Person reliability coefficient = 0.67 | ? |
|  | *Cross-cultural validity* | 7 items with DIF by cultural difference (Australian versus Taiwanese cohort) | – |
|  | *Hypotheses testing for construct validity: convergent validity* | Results not in line with 2 hypotheses  Results in line with 5 hypotheses | 2-/5+ |
|  | **CHSQ (Turkish version)** | | |
| Gün et al.(32) | *Internal consistency* | Cronbach’s alpha range = 0.83-0.86 | ? |
|  | *Reliability* | ICC range = 0.98-0.99 | + |
|  | *Hypotheses testing for construct validity: convergent validity* | Results in line with 1 hypothesis | 1+ |
|  | **DHI** | | |
| Sanal-Top et al.(43) | *Internal consistency* | Cronbach’s alpha range = 0.83-0.94 | ? |
|  | *Reliability* | ICC range = 0.84-0.93 | + |
|  | *Hypotheses testing for construct validity: convergent validity* | No hypotheses were defined a priori | ? |
|  | **HUH** | | |
| Van der Holst et al.(44) | *Reliability* | ICC = 0.89 (95% IC 0.81–0.93) | + |
|  | *Measurement error* | SEM (logits) = 0.599; SDC_individual_ (logits) = 1.66; SDC_group_ (logits) = 0.22 | ? |
|  | *Hypotheses testing for construct validity: convergent validity and discriminative validity* | Results in line with 5 hypotheses | 5+ |
|  |  |  |  |
| Geerdink et al.(35) | *Structural validity* | INFIT mean square range = 0.78-1.39; OUTFIT mean square range = 0.71-1.36 | ? |
|  | *Internal consistency* | Cronbach’s alpha = 0.941 | ? |
|  | *Hypotheses testing for construct validity: discriminative validity* | Results in line with 2 hypotheses | 2+ |
|  | **IMAL** | | |
| Carey et al.(51) | *Internal consistency* | ‘How Often’ scale: Cronbach’s alpha = 0.94  ‘How Well’ scale: Cronbach’s alpha = 0.95 | ? |
|  | *Reliability* | ‘How Often’ scale: Spearman’s correlation = 0.64  ‘How Well scale: Spearman’s correlation = 0.70 | ? |
|  | *Measurement error* | ‘How Often’ scale: SEM = 0.66  ‘How Well scale: SEM = 0.61 | ? |
|  | *Hypotheses testing for construct validity: convergent validity and discriminative validity* | No hypotheses were defined a priori | ? |
|  | **PEDI self-care domain** | | |
| Ho et al.(52) | *Hypotheses testing for construct validity: discriminative validity* | Results not in line with 1 hypothesis  Results in line with 1 hypothesis | 1-/1+ |
|  | **PODCI** | | |
| Huffman et al.(53) | *Hypotheses testing for construct validity: discriminative validity* | Results in line with 5 hypotheses | 5+ |
|  |  |  |  |
| Bae et al.(54) | *Hypotheses testing for construct validity: convergent validity and discriminative validity* | Results in line with 6 hypotheses  For several analyses hypotheses could not be defined a priori | ?/6+ |
|  |  | | |
| Dedini et al.(24) | *Responsiveness: construct approach* | Results not in line with 2 hypotheses  Results in line with 4 hypotheses | 2-/4+ |
|  |  | | |
| Squitieri et al.(50) | *Hypotheses testing for construct validity: discriminative validity* | No hypotheses were defined a priori | ? |
|  |  | | |
| Wall et al.(55) | *Hypotheses testing for construct validity: discriminative validity* | No hypotheses were defined a priori | ? |
|  | **PODCI (v2.0; Original version)** | | |
| Kunkel et al.(25) | *Internal consistency* | Cronbach’s alpha range = 0.82-0.93 | ? |
|  | *Hypotheses testing for construct validity: discriminative validity* | No hypotheses were defined a priori | ? |
|  | *Responsiveness: construct approach* | Moderate-large SRM (0.38-1.27)/effect size (0.32-1.37) for UE function, mobility, pain/comfort, happiness, global function; SRM 0.12/effect size 0.14 for sports/physical | ? |
|  | **PODCI (v2.0; Dutch version)** | | |
| Van der Holst et al.(26) | *Internal consistency* | Cronbach’s alpha range = 0.161-0.928 | ? |
|  | *Reliability* | 4 subscales and total score: ICC = 0.636–0.972 (p <0.025)  ‘Pain and comfort’-subscale: ICC = 0.022 (p = 0.476) | – |
|  | *Hypotheses testing for construct validity: convergent validity* | Results in line with 2 hypotheses | 2+ |
|  | *Responsiveness: construct approach* | No hypotheses were defined a priori | ? |
|  | **PROMIS – Upper Extremity item bank (short form)** | | |
| Waljee et al.(56) | *Hypotheses testing for construct validity: convergent validity* | Results in line with 3 hypotheses | 3+ |
|  | **PROMIS – Upper Extremity item bank (CAT)** | | |
| Waljee et al.(56) | *Hypotheses testing for construct validity: convergent validity* | Results in line with 3 hypotheses | 3+ |
|  | **QuickDASH** | | |
| Quatman-Yates et al.(57) | *Internal consistency* | Cronbach’s alpha = 0.91 | ? |
|  | *Hypotheses testing for construct validity: convergent validity* | Results in line with 1 hypothesis | 1+ |
|  | **Revised PMAL** | | |
| Wallen et al.(39) | *Structural validity* | ‘How Often’ scale: EU associated with the first PCA contrast = 2.6  ‘How Well’ scale: EU associated with the first PCA contrast = 2.5 | ? |
|  | *Internal consistency* | ‘How Often’ scale: Person reliability index = 0.90  ‘How Well’ scale: Person reliability index = 0.89 | ? |
|  | *Reliability* | ‘How Often’ scale: ICC = 0.94  ‘How Well’ scale: ICC = 0.93 | + |
|  | *Hypotheses testing for construct validity: discriminative validity* | Results in line with 2 hypotheses | 2+ |

*ICC = intraclass correlation coefficient, SEM = standard error of measurement, SDD = smallest detectable difference, LOA = limits of agreement, DIF = differential item functioning, TLI = Tucker Lewis index, CFI = Comparative fit index, RMSEA = root mean square error of approximation, SRMR = standardized root mean square residual, MDC = minimal detectable change, SDC = smallest detectable change, PCA = Principal Component Analysis, SRM = standard response mean;*

*ChARM = Children’s Arm Rehabilitation Measure, CHEQ = Children's Hand-use Experience Questionnaire, CHQ = Child Health Questionnaire, CHSQ = Children’s Hand-Skills ability Questionnaire, DHI = Duruöz Hand Index, HUH = Hand-Use-at-Home questionnaire, IMAL = Infant Motor Activity Log, PEDI = Pediatric Evaluation of Disability Inventory, PODCI = Pediatric Outcomes Data Collection Instrument, PROMIS = Patient-Reported Outcomes Measurement Information System, CAT = computer-adaptive test, DASH = Disabilities of the Arm, Shoulder and Hand, PMAL = Pediatric Motor Activity Log*

* The result of each study on a measurement property of a PROM was rated against the updated criteria for good measurement properties: – = insufficient;
+ = sufficient; ? = indeterminate.^…^

^§^ Number of hypotheses tested (2) and if the hypotheses were confirmed (+) or rejected (-) in the study
